# Green Transformational Leadership and Value–Action Barrier on Employees’ Pro-Environmental Behavior: The Moderating Role of Green Brand Image in Chinese Food Manufacturing Enterprises

**DOI:** 10.3390/bs16010071

**Published:** 2026-01-05

**Authors:** Liqing Zhong, Juhee Hahn

**Affiliations:** 1 The Graduate School, Chung-Ang University, Seoul 06974, Republic of Korea; zlqpink123@cau.ac.kr; 2 Department of Business Management, Chung-Ang University, Seoul 06974, Republic of Korea

**Keywords:** green transformational leadership, employee pro-environmental behavior, value–action barrier, green brand image, social learning theory, social cognitive theory

## Abstract

As public attention to environmental issues grows, enterprises have begun implementing environment-centered business management. Achieving environmental sustainability requires the participation of all organizational members. This study was conducted in Chinese food manufacturing small and medium-sized enterprises located in Guangdong and Jiangsu provinces, China, and employed a three-wave, time-lagged survey design to collect and match data from team leaders and employees. Hierarchical linear modeling was used to test the cross-level hypotheses, and the indirect effect was assessed using Bayesian multilevel mediation analysis. Using cross-level data from both team leaders and team members, this study examines how green transformational leadership impacts employees’ pro-environmental behavior. In addition, this study examines the mediating role of employee value–action barriers and the moderating role of green brand image. The results indicate that (1) green transformational leadership positively influences employee pro-environmental behavior, (2) employee value–action barriers mediate the relationship between green transformational leadership and employee pro-environmental behavior, and (3) green brand image moderates both the correlation between green transformational leadership and employee pro-environmental behavior and the relationship between employee value–action barriers and employee pro-environmental behavior. These findings provide empirical support for the application of social learning theory and offer managerial insights into how managers can more effectively enhance their employees’ pro-environmental behavior. Future research may further test the robustness and applicability of these relationships in other industries and in different regional and national contexts.

## 1. Introduction

Climate change is a serious global issue (Farrukh et al., 2022). Human actions and activities, such as industrial burning of coal, oil, and gas, electricity generation, and home heating, increase greenhouse gas emissions and contribute to global warming and climate change (Hanaki & Portugal-Pereira, 2018). According to the World Health Organization, climate change causes over 150,000 deaths annually, with projections indicating this will increase to 250,000 deaths yearly between 2030 and 2050. Realization of this trend has resulted in heightened environmental concerns globally, spanning both developed and developing nations. The Emissions Gap Report 2022 highlights minimal progress towards the Paris Agreement’s temperature targets since the 2021 UN Climate Change Conference. To attain the required goals by 2030, global greenhouse gas emissions must be slashed by 45% from current policy projections. Amid the significant challenges of climate change, more businesses are supporting environmental activities and practices to facilitate companies in becoming both competitive and eco-friendly (Komal S & Khandare, 2024). Research indicates that human activity significantly influences climate change; therefore, changing employee behavior is frequently cited as the most crucial step toward organizational greening (Robertson, 2018). Employees who are aware of the significance and severity of environmental issues can better respond to these challenges by adopting pro-environment behavior (PEB) to minimize resource waste and save operating costs (Farrukh et al., 2022). However, limited research has explored the determinants of employees’ pro-environmental behavior (EPB) within the context of the green transformation in the food manufacturing industry, where environment-related difficulties are particularly prominent today. This study addresses the challenges faced by Chinese food manufacturing enterprises, which contend with intense regulatory pressure, recurrent food-safety scandals, and rising consumer expectations concerning environmental and health standards. In addition, with the comprehensive advancement of China’s economic development, the food industry, as a primary driver of innovation, significantly contributes to innovative development and green development under the overarching trend of environmental protection, thereby continually promoting green and high-quality economic growth (Zhang et al., 2019). Therefore, this study adopts the green food manufacturing industry as its context, whereupon the factors that influence its EPB are examined. The findings both enlighten us and serve as the foundation for recommendations as to how to facilitate the sustainable development of the environmental protection economy.

In recent research, environmental activists and scientists have demonstrated that green and sustainable strategy implementation largely depends on leaders (Z. Li et al., 2020). The significance of leaders in influencing employee and organizational outcomes is extensively documented in the literature (A. Li et al., 2017). Therefore, certain scholars have commenced a collaborative examination of leadership and the environment, engaging in discussion on environmental leadership. Previous studies exemplified the traits of effective leaders in the environmental sector, while more recent scholars have explored the impact of leadership behavior (Afsar et al., 2016). Among many leadership models and theories, transformational leadership theory is deemed relevant to understanding environmental management because of the fact that transformational leaders are perceived to be more effective in enhancing environmental performance (Peng et al., 2021). Given this, and in line with this theme, Robertson and Barling introduced green transformational leadership (GTL) (Robertson & Barling, 2013). GTL is defined as “a form of transformational leadership that focuses on encouraging pro-environment and green initiatives.” The literature indicates sufficient evidence that GTL promotes PEB (Peng et al., 2021). According to social learning theory, EPB arises from green transformational leaders’ demonstration and influence on organization members (Pinzone et al., 2016). By supporting their organizations’ green strategies and initiatives, environmentally focused transformational leaders endeavor to communicate clear environmental values and environmental sustainability priorities to employees (Robertson & Barling, 2017), thereby developing acceptable codes of conduct and demonstrating commitment to environmental protection. By observing these leaders and learning from them, employees may perceive that their leaders prioritize environmental practices (Robertson & Barling, 2017). At the same time, when employees are supported by superior managers, they will provide feedback and participate, fostering mutual support among employee groups. This stimulates employees’ environmental awareness and encourages their PEB aimed at environment protection (Montani et al., 2017). The feedback effect of PEB within employee groups further motivates colleagues to engage in environmental protection and collaboration (Latif et al., 2022), thereby promoting positive and innovative green behaviors. Furthermore, climate strength theory, originating from Situational Strength (Yan & Hu, 2022), posits that the atmosphere and environment within an organization significantly influence employee behavior. Based on this theory, the intensity of leadership directly impacts employee behavior (Menges et al., 2011). GTL is a specific form of leadership that emphasizes leaders influencing change by fostering environmental sustainability and encouraging employees to engage in eco-friendly behaviors (Begum et al., 2022). However, limited research has explored the determinants of PEB in the context of the green transformation of the food manufacturing industry, where environment-related issues and challenges are notably significant today. To address this research gap, this study chooses EPB as the dependent variable and GTL as the independent variable to examine the impact of GTL on EPB.

In addition, EPB is influenced by both facilitating and limiting factors. Extensive research on the “value–action gap” indicates that, despite individuals endorsing pro-environmental values, various psychological and practical obstacles can hinder the translation of these values into tangible actions (Rae, 2022). Numerous organizational studies have regarded value–action barriers (VAB) as a boundary condition that diminishes the relationship between antecedents and EPB, often conceptualizing VAB as a moderating variable (Azhar & Yang, 2022). However, there is significantly less information regarding whether VAB operates as an internal psychological mechanism by which leadership shapes EPB, particularly within the framework of China’s green transformation of the food manufacturing sector. This study conceptualizes VAB as a mediating variable and investigates whether GTL can diminish VAB and consequently enhance EPB.

A green brand image (GBI) can be described as an array of ideas, beliefs, or impressions concerning a company’s environmental activities. In psychology, GBI can be divided into two parts: functional and tangible (Azhar & Yang, 2022). The GBI reflects a company’s environmental stance and distinguishes it from competitors. According to Bandura’s (1988) Social Cognitive Theory, the associated research indicates that a positive GBI can amplify the influence of GTL on EPB (Wei et al., 2023). A robust GBI heightens employees’ environmental awareness and sense of responsibility. Leaders convey the significance of environmental protection and the enterprise’s commitment via publicity, thereby enhancing their employees’ understanding of environmental issues. This heightened awareness and accountability motivate employees to transcend VAB and participate in PEB. However, empirical research on how GTL, moderated by GBI, influences EPB remains limited. Thus, this study examines GBI as a moderating variable to investigate the impact of GTL and VAB on EPB.

Against this backdrop, the goal of this study is to develop and test an integrated cross-level model that explains employee pro-environmental behavior in Chinese food manufacturing enterprises. Specifically, this study examines whether team-level green transformational leadership predicts employee pro-environmental behavior, tests value–action barriers as a mediating mechanism linking leadership to behavior, and assesses whether team-level green brand image strengthens the positive association between leadership and pro-environmental behavior and attenuates the negative association between value–action barriers and pro-environmental behavior.

Based on the above, the following questions constitute this study’s focus:

Q1: What role does GTL play in promoting EPB?
Q2: How does the VAB affect EPB?
Q3: To what extent, if any, does team-level GBI influence the relationship between (a) GTL and EPB, and (b) VAB and EPB?

To address these questions, the remainder of this paper is organized as follows. Section 2 develops the theoretical foundation and hypotheses. Section 3 describes the sample and procedure, measures, and analytic strategy. Section 4 reports the empirical results. Section 5 discusses the findings and outlines theoretical and practical implications, limitations, and directions for future research. Section 6 concludes.

## 2. Theoretical Foundation and Hypotheses Development

### 2.1. GTL and EPB

As organizations accelerate their sustainable transformation, leadership style increasingly embodies an orientation towards environmental responsibility. Transformational leadership has consistently garnered attention for its ability to stimulate employee behavior and organizational innovation (Robertson & Barling, 2013). Consequently, GTL was proposed to characterize the behavior of leaders in inspiring employees to surpass established performance goals through green values and visions (Özgül & Zehir, 2021). This leadership style prioritizes exemplifying behavior and guiding employees to integrate environmental goals into daily tasks through idealized influence, motivation, intellectual stimulation, and individualized consideration (Erkmen, 2018).

Amidst climate change and ecological constraints, enterprises need to achieve green operations through the initiative of internal members (Pereira et al., 2021). EPB is a crucial mechanism for enhancing organizational environmental performance. It encompasses energy conservation and emission reduction, resource reutilization, and advocacy of peers to adopt environmental practices (Canova & Manganelli, 2020; Kim et al., 2018). Studies indicate that leaders’ environmentally oriented behavior can markedly enhance the green participation of employees (Peng et al., 2021; Robertson, 2018). Social Learning Theory posits that employees progressively develop analogous environmental awareness and value judgments by observing and emulating the leader’s green demonstration (Pinzone et al., 2016). When leaders consistently communicate the organization’s environmental vision and foster an atmosphere of trust, employees will develop a sense of responsibility and belonging, thus establishing a positive feedback loop at the team level (Graves & Sarkis, 2018; Montani et al., 2017). In addition, Climate Strength Theory posits that the intensity of organizational context influences the consistency and direction of employee behavior (He et al., 2023; Menges et al., 2011). GTL enhances environmental protection signals and social norms within the organization, enabling employees to perpetually reinforce green behavior through social identity and group motivation (Begum et al., 2022; Weber & Kassab, 2024). Thus, we propose the following hypothesis:

**Hypothesis** **1.** *GTL positively affects EPB.*

### 2.2. GTL and VAB

Practically, employees often endorse environmental protection at the attitudinal level; however, translating such intentions into consistent day-to-day action remains challenging. This intention–behavior discontinuity is commonly described as VAB, whereby pro-environmental intentions are undermined by psychological inertia, diffusion of responsibility, and practical constraints such as insufficient time, resources, or facilities (Azhar & Yang, 2022; Blake, 1999; Gifford, 2011). Researchers further distinguish obstacles rooted in individual-level limitations (e.g., inadequate knowledge and understanding) from those arising at the societal level (e.g., insufficient institutional information and social support) (Dioba et al., 2024).

Empirical evidence suggests that leaders can mitigate employees’ behavioral barriers through value guidance and supportive organizational arrangements (Mouazen et al., 2023). Specifically, GTL may align employees with organizational environmental objectives and reduce execution inertia by cultivating a green culture and communicating a shared vision (Bronkhorst et al., 2015). From a social learning theory perspective, leaders’ consistent role modeling and feedback help employees internalize environmental responsibility and strengthen self-efficacy, thereby reducing the gap between cognition and behavior (Robertson & Barling, 2017; Saleem et al., 2021). Leaders may also stimulate green motivation and intellectual inspiration to promote process improvement and sustained green action (Çop et al., 2021; Z. Li et al., 2020). Importantly, in this study, GTL is conceptualized as a unipolar, positively valenced construct capturing the extent to which supervisors enact constructive green transformational behaviors (Ledi et al., 2024; Maitlo et al., 2022). Thus, the hypothesized “negative effect” refers to an inverse association between GTL and VAB, rather than implying a “negative form” of GTL; conceptually, concerns about self-serving or harmful “transformational” behaviors are typically discussed under pseudo-transformational leadership (Guo et al., 2025; Tian et al., 2023).Thus, hypothesis 2 follows:

**Hypothesis** **2.** *GTL negatively affects VAB.*

### 2.3. The Mediating Role of VAB Between GTL and EPB

When employees are constrained by VAB, EPB is often inhibited. The study indicated that individuals who perceive their behavior as challenging to adjust or experience excessive external pressure are likely to diminish their willingness to participate in the environmental initiatives (Hicklenton et al., 2019). In the organizational context, this psychological disparity is evident as a state of “knowledge-action inconsistency “; that is, while employees endorse the green concept, they lack practical motivation to act (Azhar & Yang, 2022).

GTL can resolve this contradiction through multiple mechanisms. By establishing explicit green standards and incentive mechanisms, leaders facilitate employees’ attainment of a sense of accomplishment and societal acknowledgement in environmental conduct (Chen & Chang, 2013). When employees encounter behavioral barriers, leaders assist them in enhancing their green innovation capabilities and goal persistence through intellectual motivation and value augmentation (Ryan & Deci, 2000). This reciprocal influence increases employees’ internal motivation while diminishing external resistance, thereby increasing their propensity to translate environmental intentions into tangible actions. Consequently, we propose the subsequent hypothesis:

**Hypothesis** **3.** *VAB will mediate the relationship between GTL and EPB.*

### 2.4. The Moderating Role of GBI Between GTL and EPB

The intensification of global environmental problems has prompted enterprises to integrate environmental responsibility into the foundation of their strategies. GBI embodies the company’s environmental commitment and reputation among the public and employees (Morss et al., 2011; Muisyo et al., 2022). Organizations possessing a positive brand reputation typically prioritize green innovation in production design, supply chain management, and waste treatment (Begum et al., 2022; Xie et al., 2019). An effective brand image improves external competitiveness and enhances employees’ alignment with the corporate mission, thus stimulating their green behavior (Sujanska & Nadanyiova, 2023).

Social Cognitive Theory posits that GBI can strengthen the impact of GTL on employee behavior (Bandura, 1988; Khan et al., 2021). When employees recognize the genuine enterprise’s investment in environmental protection, they are more inclined to view the leader’s statements and actions as credible exemplars, forming a positive organizational climate. Research indicates that GBI can amplify the influence of leadership behavior on employees’ environmental responsibility and propensity to act (Zhou et al., 2021). However, in the domain of human resources and organizational behavior, studies on this adjustment mechanism remain relatively limited, especially at the employee level. Therefore, we propose the following hypothesis:

**Hypothesis** **4.** *GBI will moderate the relationship between GTL and EPB.*

### 2.5. The Moderating Role of GBI Between VAB and EPB

When employees struggle to engage in environmentally friendly behaviors due to time constraints, lack of motivation, or inherent habits, external cues within the organization merge as critical influencing factors (Kollmuss & Agyeman, 2002). Research indicates that harmonizing personal values with the organization’s brand identity can significantly promote behavioral change (Foster et al., 2022). A favorable GBI can strengthen employees’ sense of belonging and recognition, thereby increasing their willingness to participate in environmental protection initiatives (Ateş, 2020; Cheng et al., 2021; Saifulina et al., 2023).

According to Social Cognitive Theory, brand image offers employees a definitive expectation framework and social relevance (Erkmen, 2018). When employees comprehend and endorse the environmental value embodied by the brand, they will inherently uphold this image and exhibit an elevated sense of responsibility in their actions (Burmann & Zeplin, 2005; Löhndorf & Diamantopoulos, 2014). This social identity mechanism motivates employees to overcome inertia and personal limitations, viewing environmental protection behavior as integral to collective norms and self-expression (Yang et al., 2022). When employees perceive themselves as members of a larger family, they are more inclined to engage in collective environmental efforts. This social identification and collective consciousness assist them in surmounting temporal limitations and personal hurdles, facilitating active involvement in environmental initiatives. Therefore, the subsequent hypothesis is posited:

**Hypothesis** **5.** *GBI will moderate the relationship between VAB and EPB.*

**Figure 1:**
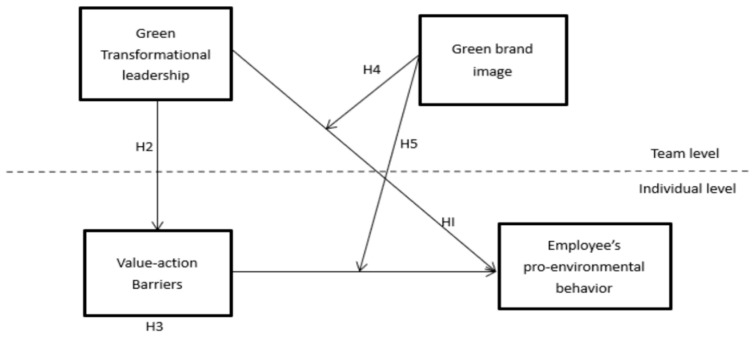
Theoretical Framework.

To summarize the theoretical framework of the present study, Figure 1 depicts the proposed research model and the hypothesized relationships among green transformational leadership, the value–action barrier, green brand image, and employee pro-environmental behavior across the team and individual levels.

## 3. Methods

### 3.1. Sample and Procedure

This study collected survey data from Chinese food manufacturing enterprises. Prior to implementation, we contacted the human resources (HR) departments of participating firms. After obtaining organizational permission from HR directors, an HR contact served as the liaison and coordinated questionnaire distribution. Team leaders were asked to distribute employee questionnaires to their direct subordinates and return completed questionnaires to the research team via the HR contact.

To enhance construct validity and reduce common method variance, we employed a multilevel, three-wave time-lagged design and clearly specified which respondent rated each construct at each wave (Podsakoff et al., 2003). All questionnaires were self-administered and completed individually rather than in a group setting. To minimize coworker discussion and potential social desirability pressure, participants were instructed to complete the survey privately and refrain from sharing or discussing questionnaire items with others. Prior to completing the Time 1 (T1) survey, each participant received a written information sheet describing the study purpose, the voluntary nature of participation, confidentiality protections, and the right to withdraw at any time without penalty; informed consent was obtained at this point (i.e., before any responses were provided). At the beginning of Times 2 and 3, the same confidentiality and voluntariness information was reiterated before participants completed the follow-up questionnaires.

The participating firms were small and medium-sized enterprises (SMEs), including eight SMEs from Guangdong Province and nine SMEs from Jiangsu Province. Data were collected from 25 March to 28 April 2023. At Time 1 (T1; 25 March–2 April 2023), employees evaluated the green transformational leadership (GTL) of their immediate supervisors. At Time 2 (T2; 9–15 April 2023), employees reported their value–action barriers (VAB), while team leaders simultaneously rated the green brand image (GBI) of their teams. At Time 3 (T3; 22–28 April 2023), employees reported their pro-environmental behavior (EPB). This design enabled temporal separation of key variables and a clear linkage between employee- (Level 1) and leader- (Level 2) data.

To match leader and employee questionnaires at the team level, the HR contact assigned each participating team a unique team code and prepared separate questionnaire packets for team leaders and team members. The same team code was printed on all questionnaires within a given team. To match employees’ responses across the three waves without collecting names, each employee used an anonymous respondent code consistently at T1–T3. After data collection, we first matched employees’ surveys across waves using respondent codes and then linked employees to their corresponding team leader using the shared team code. Team-level GTL was operationalized by aggregating employees’ T1 ratings within each team, and leader-rated GBI was linked to all employees within the same team for the multilevel analyses.

Given that respondents were Chinese, we ensured linguistic and conceptual equivalence of the measures using a translation and back-translation procedure consistent with established cross-cultural adaptation guidelines (Lei et al., 2022). The questionnaire was initially developed in English, translated into Chinese, and then back-translated into English by two bilingual researchers. Discrepancies were resolved through discussion until conceptual consistency was achieved. The final instrument included measures of GTL, EPB, GBI, and VAB. The informed consent form and the questionnaire (English translation) are provided in the Supplementary Materials (Files S1 and S2).

Table 1 summarizes the respondents’ demographic characteristics. The sample comprised 88 team leaders and 523 team members. Among leaders, 58.0% (N = 51) were male and 42.0% (N = 37) were female; among members, 56.4% (N = 295) were male and 43.6% (N = 228) were female. Most leaders were aged 36–50 years (86.4%, N = 76). Team members were more evenly distributed across age groups: 20–35 years (30.2%, N = 158), 36–50 years (37.9%, N = 198), and 51 years or above (31.9%, N = 167). Regarding education, 89.8% of leaders (N = 79) possessed a bachelor’s degree, while 6.8% (N = 6) held a graduate degree or higher. Among the members, 38.0% (N = 199) possessed a bachelor’s degree, 34.4% (N = 180) held a junior-college degree or lower, and 27.5% (N = 144) attained a graduate degree or higher. Regarding monthly income, the predominant category for leaders was 8000–15,000 Chinese renminbi (RMB) (38.6%, N = 34), while for team members it was 15,000 RMB or above (40.5%, N = 212). For team size, 72.7% of teams (N = 64) consisted of 11–20 members, 8.0% (N = 7) included 1–10 members, and 19.3% (N = 17) contained 21 or more members. These distributions reflect a demographically diverse sample appropriate for comparing team leaders and team members regarding the focal study variables.

### 3.2. Measures

Unless otherwise indicated, all scale items were assessed using a five-point Likert-type response format (1 = “strongly disagree” to 5 = “strongly agree”). Composite scores were computed by averaging the corresponding items, with higher values indicating higher levels of the focal construct.

#### 3.2.1. Green Transformational Leadership

The six-item scale created by Chen and Chang (2013) assesses leaders’ effectiveness in conveying and inspiring a green vision. The primary questions encompass: “My superiors actively promote the concept of environmental protection in daily management”, “My superiors encourage team member’s to participation in green innovation initiatives”, “My superiors exemplify environmentally protective behaviors”, “My superiors often commend employees who excel in environmental protection”, “My superiors establish explicit goals pertaining to environmental protection”, “My superiors prioritizes resource conservation and reutilization in their work”. Item responses were averaged, with higher scores indicating higher perceived GTL. Internal consistency was high (Cronbach’s alpha = 0.944).

#### 3.2.2. Employees’ Pro-Environmental Behavior

The seven-question scale developed by Frese et al. (1997) is used to assess employees’ green behavior at work. The inquiries encompass: “I will proactively initiate efforts to minimize resource waste at work”, “I will actively endorse the company’s environmental protection initiatives “, “I often remind my colleagues to conserve electricity and water”, “I am willing to try new environmental protection practices”, “I am open to experimenting with new environmental protection practices”, “I will propose enhancements to process aimed at pollution reduction, “I am prepared to assume additional responsibilities for achieving the company’s environmental objectives”. Item responses were averaged, with higher scores indicating higher EPB. Internal consistency was high (Cronbach’s alpha = 0.937).

#### 3.2.3. Value–Action Barrier

Utilizing the framework established by Lorenzoni et al. (2007) to assess the psychological and practical obstacles of employees in bridging value identification and action implementation. The principal enquiries include: “I regard environmental protection is crucial, yet I lack sufficient time to practice”, “Environmental protection actions require excessive energy”, “I aspire to protect the environment, but my peers do not endorse it”, “Inadequate environmental protection infrastructure hinders my ability to act”, “I’m concerned that environmental protection behavior may affect work performance”, “I believe my individual action have minimal impact on the environment.” Item responses were averaged, with higher scores indicating stronger perceived barriers. Internal consistency was acceptable (Cronbach’s alpha = 0.893).

#### 3.2.4. Green Brand Image

Zameer et al. (2020)proposes a 5-item scale to assess employees’ perception of the company’s environmental protection image. The topics include: “I believe the company possesses a commendable reputation in environmental protection”, “The company’s products demonstrates the commitment to environmental protection”, “The company is an industry leader in green production and packaging”, “The company actively communicates the concept of sustainable development to the public”, “I perceive the company as having a positive image regarding environmental protection”. Item responses were averaged, with higher scores indicating a more favorable perceived green brand image. Internal consistency was acceptable (Cronbach’s alpha = 0.849).

#### 3.2.5. Questionnaire Validation

To document the psychometric quality of the questionnaire, we followed commonly recommended measurement validation procedures. Internal consistency reliability was assessed using Cronbach’s alpha, with values of 0.70 or higher indicating acceptable reliability (Youssef et al., 2023); the alpha coefficients for each scale are reported in Section 3.2.1, Section 3.2.2, Section 3.2.3 and Section 3.2.4. Construct validity was examined via confirmatory factor analysis (CFA). Model fit was evaluated using multiple indices, including the comparative fit index (CFI) and Tucker–Lewis index (TLI) (≥0.90) as well as the root mean square error of approximation (RMSEA) and standardized root mean square residual (SRMR) (≤0.08) (Morales Chainé et al., 2022). Convergent validity was assessed using standardized factor loadings, composite reliability (CR ≥ 0.70), and average variance extracted (AVE ≥ 0.50) (Fornell & Larcker, 1981). Discriminant validity was evaluated using the Fornell–Larcker criterion, whereby the square root of AVE for each construct should exceed its correlations with other constructs (Fornell & Larcker, 1981). The empirical results of these measurement assessments are reported in Section 4.

### 3.3. Analytic Strategy

Given the nested data structure (employees nested within teams), analyses were conducted in a staged manner that mirrors the sequence of results reported in Section 4. First, we screened the data and computed descriptive statistics (including frequency distributions for demographic variables, means, standard deviations, and zero-order correlations) using SPSS 26.0. Second, to diagnose potential common method variance, we compared CFA models with and without an unmeasured latent method factor and inspected changes in fit indices following recommended guidelines (Podsakoff et al., 2003). Third, we evaluated the measurement model in Mplus 8.3 via confirmatory factor analysis (CFA), comparing the hypothesized four-factor model with plausible alternative models and assessing global fit using multiple indices (Choisay et al., 2021). Convergent validity was assessed through standardized loadings, composite reliability, and average variance extracted, and discriminant validity was assessed using the Fornell–Larcker criterion (Anderson & Gerbing, 1988; Fornell & Larcker, 1981).

Fourth, hypotheses were tested using two-level hierarchical linear modeling in Mplus 8.3 with full maximum likelihood estimation. We estimated unconditional (null) models to partition within- and between-team variance, entered control variables at the employee level (Level 1) and team level (Level 2), and then tested the cross-level direct effects of team-level GTL on employee EPB (H1) and the team-level effect of GTL on employee VAB (H2). Consistent with multilevel modeling recommendations, Level-1 predictors were group-mean centered and Level-2 predictors were grand-mean centered to facilitate interpretation of within-team and between-team effects (Enders & Tofighi, 2007). Fifth, we evaluated the indirect effect of team-level GTL on EPB via VAB (H3) using Bayesian multilevel mediation with credibility intervals (Preacher et al., 2010). Finally, cross-level moderation hypotheses were tested by adding interaction terms for GTL × GBI (H4) and VAB × GBI (H5), and significant interactions were probed using simple-slope analyses at high (+1 SD) and low (−1 SD) levels of GBI (Preacher et al., 2006).

## 4. Results

All statistical analyses were conducted using SPSS 26.0 and Mplus 8.3. Specifically, descriptive statistics and preliminary analyses (e.g., frequency distributions) were performed in SPSS 26.0, whereas confirmatory factor analyses and the subsequent multilevel hypothesis tests (including the Bayesian multilevel mediation model) were estimated in Mplus 8.3.

### 4.1. Common Method Variance Test

This study first examined common method bias through a single-factor confirmatory factor analysis (CFA). All items relevant to the hypothesis testing were assigned to one latent factor. If the single-factor model demonstrates substantially inferior fit to the original measurement model, common method bias is unlikely to be a substantial concern. The CFA results indicated that the single-factor model exhibited a markedly poorer fit than the original four-factor model. Specifically, as shown in Table 2, the four-factor model displayed good fit (χ^2^/df = 1.537, CFI = 0.983, TLI = 0.981, RMSEA = 0.032, SRMR = 0.031), whereas the single-factor model revealed poor fit (χ^2^/df = 19.348, CFI = 0.407, TLI = 0.350, RMSEA = 0.187, SRMR = 0.194). The chi-square difference test was significant at the 0.001 level, suggesting that serious common method bias is improbable.

In addition, this study employed a CFA model incorporating a latent common-method factor to assess common-method bias. All items were designed to load on both their respective theoretical constructs and the common-method factor. If the model with the common-method factor does not significantly improve model fit, specifically, if changes in RMSEA and SRMR are within 0.05 and changes in CFI and TLI are within 0.10, then common method variance may be considered negligible. Table 2 illustrates that the comparison between the model with the common-method factor and the original four-factor model revealed minimal changes in the key fit indices (ΔCFI = 0.002, ΔTLI = 0.003, ΔRMSEA = 0.002, ΔSRMR = 0.000; SRMR remained 0.031 in both models), all below 0.05. These findings indicate that adding the common-method factor did not significantly improve model fit, suggesting that common method bias is not a serious concern in this study (Podsakoff et al., 2003).

### 4.2. Confirmatory Factor Analysis

Using Mplus 8.3, we conducted a confirmatory factor analysis to assess the distinctiveness of variables. We assessed model fit using the overall chi-squared statistic, root mean square error of approximation, the comparative fit index, the goodness-of-fit index, and the Tucker–Lewis index. The findings indicate that the four-factor model (GTL, EPB, VAB, GBI) exhibits superior fit compared to the three-factor model (GTL + GBI, EPB, VAB), the two-factor model (GTL + GBI + VAB, EPB), and the one-factor model (GTL + GBI + VAB + EPB). Therefore, the four-factor model is considered the most appropriate for this study.

Table 3 reports the convergent validity indicators for all constructs. Standardized factor loadings were substantial (0.601–0.879), and composite reliability (CR) values exceeded 0.70, while average variance extracted (AVE) values exceeded 0.50, supporting adequate convergent validity (Fornell & Larcker, 1981; Anderson & Gerbing, 1988). Overall, these results indicate that the measurement model exhibits satisfactory convergent validity for subsequent analyses.

### 4.3. Correlation Analysis

Table 4 reports the means, standard deviations, and correlations for all variables at both the individual and team levels. At the individual level, the mean scores were 3.325 (SD = 0.883) for EPB and 3.752 (SD = 0.843) for VAB, with the control variables also exhibiting moderate dispersion (e.g., age: M = 2.020, SD = 0.789; income: M = 2.100, SD = 0.837). At the team level, the mean score for GTL was 3.590 (SD = 0.848) and for GBI was 3.485 (SD = 0.712), and the average team size fell between 11 and 20 members (M = 2.120, SD = 0.506). Most zero-order correlations were in the anticipated directions: individual EPB was negatively correlated with VAB (r = −0.278, *p* < 0.001) and income (r = −0.140, *p* < 0.01), whereas team-level GTL was positively correlated with GBI (r = 0.591, *p* < 0.001) and team size (r = 0.306, *p* < 0.001). In addition, discriminant validity was evaluated using the Fornell–Larcker criterion. For each construct, the square root of its AVE (diagonal elements) exceeded its correlations with other constructs, indicating that each construct captured more variance in its indicators than it shared with other constructs and thus supporting satisfactory discriminant validity (Fornell & Larcker, 1981).

### 4.4. Hypothesis Tests

Based on the hierarchical linear modeling results presented in Table 5, several patterns emerged that align with the proposed hypotheses. In Null Model 1, only the intercept for employee pro-environmental behavior (EPB) was estimated (γ = 3.320, *p* < 0.001). In Model 1, individual- and team-level control variables were incorporated; none of these controls indicated a significant effect on EPB, except for team size (γ = 0.522, *p* < 0.001). When green transformational leadership (GTL) was entered at the team level in Model 2, GTL exerted a significant positive effect on EPB (γ = 0.506, *p* < 0.001), thereby supporting Hypothesis 1.

To test the mediating role of value–action barrier (VAB), Model 3 simultaneously included GTL and VAB. VAB was negatively correlated with EPB (γ = −0.241, *p* < 0.001), and the coefficient for GTL decreased from 0.506 (*p* < 0.001) in Model 2 to 0.444 (*p* < 0.001) in Model 3, thereby indicating a reduction in the direct effect while remaining significant. These results align with a partial mediation pattern, thus supporting Hypothesis 3.

The effects of GTL on VAB were examined in the right-hand part of Table 4. In Null Model 2, only the intercept for VAB was significant (γ = 3.760, *p* < 0.001). After incorporating predictors in Model 5, GTL exhibited a significant negative association with VAB (γ = −0.266, *p* < 0.001), thus supporting Hypothesis 2.

The moderating role of green brand image (GBI) was tested in Models 4 and 5. In Model 4, both the main effect of GBI (γ = 0.205, *p* < 0.05) and the interaction term GTL × GBI (γ = 0.466, *p* < 0.001) were significant, indicating that the positive relationship between GTL and EPB is more significant when GBI is higher; this finding supports Hypothesis 4. In Model 5, the interaction between GBI and VAB (GBI × VAB) was also significant and positive (γ = 0.359, *p* < 0.01), indicating that the effect of VAB varies as a function of GBI, thereby supporting Hypothesis 5.

### 4.5. Mediation Analysis

To assess whether VAB mediates the effect of GTL on EPB, a mediation model was estimated using a Bayesian approach, suitable for multilevel data with a limited number of clusters. Table 6 presents the posterior estimates. The posterior mean of the direct effect of GTL on EPB was 0.407, and the 95% credible interval [0.251, 0.561] excluded zero, indicating a robust positive direct correlation between GTL and EPB. The indirect effect of GTL on EPB via VAB was 0.066, with a 95% credible interval [0.024, 0.121] excluding zero. This suggests that increased GTL correlates with decreased VAB, subsequently predicts higher EPB. The significant difference from zero in both the direct and indirect effects indicates partial mediation, thereby corroborating Hypothesis 3.

### 4.6. Moderation Analysis

**Figure 2:**
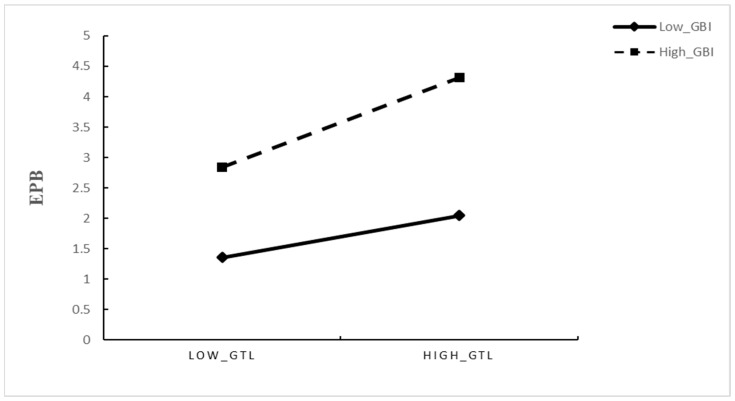
Effect of Green transformational Leadership × Green brand image on Employer pro-environmental behaviors.

An interaction term (GTL × GBI) was included in the model to assess GBI’s moderating effect on the relationship between GTL and EPB. The interaction was significant and positive (B = 0.466, *p* < 0.001), and the main effect of GTL on EPB was also significant and positive (B = 0.407, *p* < 0.001), indicating that GBI strengthens the positive impact of GTL on EPB. Simple-slope analyses utilizing high and low levels of GBI (±1 SD) further elucidated this pattern (see Table 7 and Figure 2). When GBI was high, GTL exerted a strong positive effect on EPB (B = 0.873, 95% CI [0.647, 1.102], *p* < 0.001). Conversely, when GBI was low, the effect of GTL on EPB was not significant (B = −0.061, 95% CI [−0.262, 0.140], *p* > 0.05). The difference between the two simple slopes was significant (ΔB = 0.932, 95% CI [0.635, 1.247], *p* < 0.001), demonstrating that employees’ perceptions of a strong green brand image amplify the positive association between GTL and EPB. These results substantiate Hypothesis 5.

**Figure 3:**
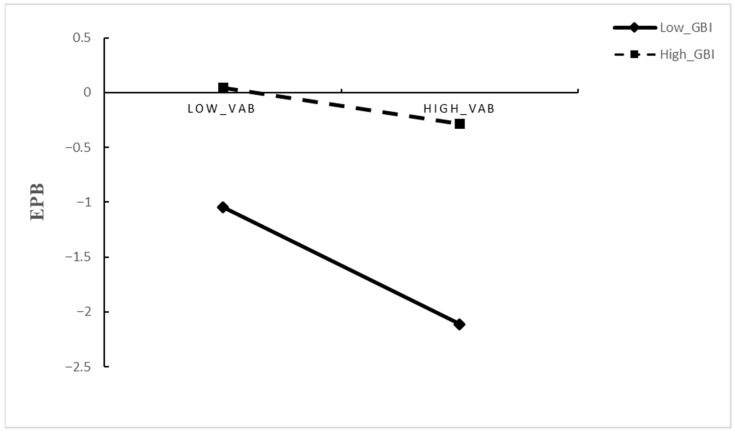
Effect of Value–Action Barrier × Green brand image on Employee pro-environmental behavior.

An interaction term (VAB × GBI) was incorporated into the model to assess whether GBI moderates the relationship between VAB and EPB. The interaction was significant and positive (B = 0.359, *p* < 0.001), whereas the primary effect of VAB on EPB persisted as significantly negative (B = −0.252, *p* < 0.001), suggesting that GBI attenuates the negative impact of VAB on EPB. Simple-slope analyses at high and low levels of GBI (±1 SD) further clarified this pattern (see Table 8 and Figure 3). At elevated GBI levels, the relationship between VAB and EPB was not significant (B = 0.108, 95% CI [−0.123, 0.333], *p* > 0.05). In contrast, at low GBI levels, VAB exerted a significant negative effect on EPB (B = −0.612, 95% CI [−0.838, −0.384], *p* < 0.001). The disparity between these two simple slopes was significant (ΔB = 0.717, 95% CI [0.286, 1.142], *p* = 0.001), indicating that a stronger green brand image mitigates the adverse effect of value–action barriers on employees’ pro-environmental behavior. These results support Hypothesis 6.

## 5. Discussion

### 5.1. Theoretical Implications

This study enhances the literature on green organizational behavior by employing a cross-level approach to connect GTL, VAB, and EPB, with GBI as a contextual moderator. The results advance beyond simple replication of prior findings and address significant controversies in this domain: the suitable analysis level for examining green leadership effects and the mechanisms through which they manifest. The findings particularly highlight a multi-level framework by which leadership and internal branding collaboratively influence employee sustainability behaviors, thereby integrating previously distinct ‘barrier’ and ‘mechanism’ perspectives.

First, GTL at the team level exerts a significant positive effect on EPB at the individual level (supporting false Set up H1). This outcome aligns with previous literature and simultaneously broadens the scope of research. Previous studies have focused on the impact of leaders on employees’ non-green performance or psychological results. This study reveals how leaders can promote employees to adopt sustainable behavior through green value guidance and social demonstration (Ahmad et al., 2021). This study further corroborates that EPB results from the social learning process influenced by GTL, as posited by Bandura’s Social Learning Theory (Pinzone et al., 2016). This study employs a cross-layer design to elucidate the transmission pathway of team leadership behavior to individual employee behavior, thereby enriching the multi-level theoretical framework linking green leadership and employee behavior, contrary to prior research that validated only at a single level. This discovery, combined with the food manufacturing industry’s background, demonstrates that the leadership’s green value orientation can be translated into specific employee behavior across production, packaging, and quality management, thereby enhancing alignment of the green supply chain and safe production practices.

Second, employee-level VAB plays a significant mediating role between GTL and EPB (supporting Hypothesis 3). Prior research has often treated VAB as a moderating factor (Azhar & Yang, 2022); however, the present study conceptualizes VAB both as an antecedent and as a mediating mechanism, thereby extending the theoretical scope of this construct. The results suggest that when employees experience pressure stemming from workplace environmental demands, their green behaviors in contexts outside the organization may decline (Francoeur et al., 2021). GTL can help employees overcome psychological barriers and behavioral inertia by providing value-based guidance and organizational support, thereby fostering higher levels of green practice. This mechanism-based contribution also complements sustainability research that emphasizes structural and technical pathways to emission reduction. For instance, evidence from urban and residential sustainability indicates that technical energy-efficiency measures can facilitate carbon reduction, yet their successful implementation and sustained effects often still depend on complementary behavioral routines, organizational coordination, and follow-through in execution (Bragança & Verde Muniesa, 2023). Accordingly, the present study adds value by specifying a behavioral and leadership-based explanatory pathway that can operate in parallel with technical interventions.

Third, GBI significantly moderates the relationship between GTL and EPB, thereby supporting Hypothesis 4. Prior research has largely emphasized brand image in the consumer domain (Papista & Dimitriadis, 2019); in contrast, the present study demonstrates—from an organizational perspective—that brand image also amplifies the behaviors of internal stakeholders. Moreover, it connects leadership research to evidence from broader food-related contexts, where reputation- and brand-related cues shape stakeholders’ evaluations and actions. For example, research on food-delivery services suggests that corporate reputation is jointly shaped by multiple drivers and that sustainability-related considerations can meaningfully influence reputational evaluations (Dospinescu et al., 2020). In line with Bandura’s (1988) social cognitive theory, a positive brand image can heighten employees’ environmental awareness and strengthen their action identity, thereby reinforcing the transmission of leadership behaviors (Wei et al., 2023). Taken together, these findings indicate that when food manufacturing enterprises possess a strong GBI, employees are more likely to enact green commitments in their day-to-day production and managerial practices, thereby sustaining the firm’s environmental reputation through concrete behaviors.

Finally, GBI also moderates the relationship between VAB and EPB, supporting Hypothesis 5. Prior research suggests that brand image provides employees with a salient reference point for organizational expectations and value orientation (Erkmen, 2018). The present study further demonstrates that this function is particularly important for overcoming behavioral barriers. When employees strongly align with the organization’s sustainability values and brand mission, they are more likely to transcend constraints related to time, resources, and motivation and to engage proactively in green practices such as energy conservation, emission reduction, and waste management. This implies that an effective GBI can strengthen organizational identification and a sense of belonging, attenuate the detrimental effect of VAB, and promote the persistence of individual green actions. Interpreting this moderating effect against a broader body of evidence is also informative: in food markets, consumers’ perceptions and behavioral intentions are shaped by communication strategies and social media characteristics. For instance, research on healthy food marketing indicates that social media and influencer-related strategies can significantly affect consumers’ perceptions and purchase decisions (Miquel Vidal & Castellano-Tejedor, 2022). Extending this logic inward, the current findings suggest that brand-related meaning operates not only for consumers but also within organizations, where it can function as an internal climate cue that supports employees’ enactment of green behaviors even when perceived barriers are salient.

The study’s primary hypotheses have been corroborated by the aforementioned analysis. GTL improves the employees’ green behavior both directly and indirectly through the mediating effects of VAB and GBI. The adjustment mechanism establishes multi-tiered influence pathways. These results are particularly relevant in the food manufacturing industry: the green orientation of leaders, the evolution of employees’ values, and the brand’s ecological commitment collectively constitute a significant impetus for advancing the green transformation and sustainable production within enterprises.

### 5.2. Practical Implications

First, this study elucidates the significance of employees’ pro-environmental behaviors within organizations. Similar findings have been reported by Zientara and Zamojska (2018). In the workplace, employee learning allows organizations to transcend the limitations of existing knowledge resources. This suggests that a transformational leadership style is inadequate. To effectively attain the green product innovation goals, it is essential to cultivate autonomous and prosocial environmental behaviors among employees (Liu et al., 2022). Organizations must prioritize employee awareness of green product innovation objectives and recognize that environmental protection is a critical organizational goal (Polas et al., 2023). It is imperative to explicitly delineate the environmental benefits and obligations that employees ought to receive. We recommend that manufacturing companies emphasize integrating green innovation into organizational policies, practices, and procedures, thereby creating favorable conditions that inspire environmentally sustainable behaviors in employees.

Second, companies can enhance EPB by improving the work environment to reduce the VAB experienced by employees. Effective strategies include ample parking availability, flexible schedules, and informative charts on alternative transportation benefits. Implementing voice-controlled lighting in restrooms enhances convenience and environmental efficiency. To enhance ecological initiatives, managers can optimize paperwork processes and collect ideas during staff meetings to minimize paper usage.

Third, compared with firms that lack a robust green brand image (GBI), those with a strong GBI are more likely to achieve market success when introducing new green products (Zameer et al., 2020). This argument is also consistent with evidence from food-related contexts showing that food reputation and brand/packaging cues can shape stakeholders’ evaluations and behavioral intentions (Bonaiuto et al., 2012; Kunz et al., 2020; Vermeir & Roose, 2020). Accordingly, leaders should encourage marketing managers to develop coherent green marketing strategies that strengthen employees’ perceptions of the firm’s environmental commitment. Firms should also re-evaluate their production approaches and prioritize environmentally sustainable practices to enhance GBI. More broadly, manufacturing enterprises should incorporate environmental considerations throughout the product life cycle, provide environmentally friendly products, prioritize green innovation, and explicitly emphasize sustainable development.

Fourth, leaders must acknowledge their crucial role in motivating, developing, and fostering employees’ green behavior and values, particularly within the food industry (Sobaih et al., 2022). Managers should develop strategies to improve employees’ environmental behavior. Leaders should engage employees in dialogues about eco-friendly practices, exemplifying pro-environmental behaviors, and inspire others to emulate those actions. Organizations must establish environmental performance objectives and promote a culture of transparent communication, offering guidance to employees and training management to achieve these goals. Furthermore, leaders can facilitate training programs and workshops to augment employees’ environmental knowledge (Graves & Sarkis, 2018).

### 5.3. Limitation and Future Research Direction

First, previous research has predominantly treated the green brand image as a variable at the organizational level. However, due to the study’s primary emphasis on teams and its restricted scope, the team was designated as the unit of analysis, and questionnaires were distributed accordingly. Therefore, future research may be undertaken at the organizational level to further explore this phenomenon.

Second, EPB literature has delineated various forms of pro-environmental behavior that may differ in cognitive simplicity or complexity (Colombo et al., 2023), difficulty or ease, and necessity or voluntariness (Uren et al., 2021). Future research may delve deeper into EPB to comprehensively investigate its relationship with public organizational factors and individual characteristics of public employees.

Third, despite this study employing a three-wave, time-lagged, multi-source design (assessing GTL at T1, VAB, and GBI at T2, and EPB at T3) to mitigate common-method bias and strengthen evidence for temporal precedence, the data remain fundamentally non-experimental and thus cannot substantiate strong causal claims. Temporal separation and procedural remedies can alleviate, but not completely eradicate, concerns about common method variance and endogeneity, including risks such as omitted variables, reverse causality, and unobserved confounders (Podsakoff et al., 2003; Maxwell & Cole, 2007). Future research should utilize stronger causal identification strategies, including cross-lagged panel models, longitudinal field or quasi-experimental designs, or instrumental-variable approaches, to rigorously evaluate the directional assumptions inherent in the proposed GTL–VAB–EPB framework.

The study primarily involves leaders and employees within the green food sector, thereby lacking the diversity that would arise from examining companies with varied backgrounds. Every organization may possess distinct business models and environmental viewpoints. Additionally, cultural differences between Western and Chinese societies may affect levels of environmental awareness among leaders and employees. Future research may expand its scope to include companies outside mainland China and analyze frameworks across countries so as to enhance understanding of national contexts and cultural differences. Analyzing additional sectors such as pharmaceuticals, automotive, and agriculture in China and other regions would corroborate and contrast the study’s findings. This study focused on small and medium-sized enterprises; however, future research could contrast them with large corporations to assess the impact of company size on leadership (Khan et al., 2021).

## 6. Conclusions

This study examined how GTL shapes EPB in Chinese food manufacturing enterprises, with a focus on employees’ VAB and team level GBI. Based on three-wave, time-lagged data from matched leaders and employees and hierarchical linear modeling combined with Bayesian mediation analysis, the results show that GTL at the team level is positively related to EPB at the individual level. Moreover, VAB partially mediates this relationship, suggesting that GTL not only provides vision and role modeling but also helps employees to overcome psychological and practical obstacles that hinder the translation of pro-environmental values into concrete actions. In addition, GBI at the team level strengthens the positive association between GTL and EPB and weakens the negative impact of VAB on such behavior.

Overall, these findings indicate that leadership, internal psychological barriers, and brand-related context jointly shape everyday pro-environmental behavior at work in a highly regulated and reputation-sensitive sector such as food manufacturing. For managers, the results imply that strengthening GTL, systematically reducing VAB, and building a credible GBI can serve as complementary levers to foster more sustainable behavior in daily operations.

## Figures and Tables

**Table 1:** Descriptive characteristics of the sample.

Variable	Category	Team Leaders		Employees Demographic	
		Frequency	Percentage (%)	Frequency	Percentage (%)
Gender	Male	51	58	295	56.4
	Female	37	42	228	43.6
Age	20–35 years	2	2.3	158	30.2
	36–50 years	76	86.4	198	37.9
	51 years and above	10	11.4	167	31.9
Educational level	Junior college or below	3	3.4	180	34.4
	Bachelor’s degree	79	89.8	199	38
	Graduate degree or above	6	6.8	144	27.5
Monthly income	Less than 8000	21	23.9	159	30.4
	8000–15,000	34	38.6	152	29.1
	15,000 or above	33	37.5	212	40.5
Team size	1–10 people	7	8	-	-
	11–20 people	64	72.7	-	-
	More than 21 people	17	19.3	-	-

Note: Exact age data were not collected.

**Table 2:** Results of confirmatory factor analyses.

Model	χ^2^	df	χ^2^/df	RMSEA	CFI	TLI	SRMR
Four-factor model: GTL, EPB, VAB, GBI	378.11	246	1.537	0.032	0.983	0.981	0.031
Three-factor model: GTL + GBI, EPB, VAB	1099.234	249	4.415	0.081	0.891	0.879	0.074
Two-factor model: GTL + GBI + VAB, EPB	2549.150	251	10.156	0.132	0.705	0.676	0.148
One-factor model: GTL + GBI + VAB + EPB	4875.718	252	19.348	0.187	0.407	0.350	0.194
Common Method Factor	401.357	241	1.665	0.034	0.981	0.978	0.031

Note: “+” represents two factors merged into one. Abbreviations: χ^2^, Chi-square statistic; df, degree of freedom; CFI, comparative fit index; RMSEA, root-mean-square error of approximation; TLI, Tucker–Lewis index; GTL = Green Transformational Leadership; EPB = Employees’ pro-environmental behavior; VAB = Value–Action Barrier; GBI = Green Brand Image.

**Table 3:** Scales’ reliability and validity.

Variable	Items	Factor Loading	CR	AVE
Green Transformational Leadership	6	0.841~0.879	0.944	0.737
Employees’ pro-environmental behavior	7	0.816~0.837	0.937	0.680
Value–Action Barrier	6	0.688~0.822	0.893	0.583
Green Brand Image	5	0.601~0.878	0.851	0.536

Note: CR = Composite reliability; AVE = Average variance extracted.

**Table 4:** Means, Standard Deviations, and Correlations of Variables Studies.

	Mean	SD	1	2	3	4	5	6	7
Individual level									
1. Gender	1.440	0.496	1						
2. Age	2.020	0.789	−0.004	1					
3. Education	1.930	0.785	0.032	0.664 ***	1				
4. Income	2.100	0.837	−0.050	−0.030	0.071	1			
5. EPB	3.325	0.883	−0.027	−0.030	−0.055	−0.140 **	1		
6. VAB	3.752	0.843	0.040	−0.016	−0.004	−0.035	−0.278 ***	1	
Team level									
1. Gender	1.420	0.494	1						
2. Age	2.090	0.354	−0.029	1					
3. Education	2.040	0.317	0.124 **	0.255 ***	1				
4. Income	2.140	0.774	0.176 ***	−0.187 ***	−0.112 *	1			
5. Size	2.120	0.506	−0.095 *	0.129 **	−0.028	0.166 ***	1		
6. GTL	3.590	0.848	−0.081	0.116 **	0.102 *	−0.163 ***	0.306 ***	1	
7. GBI	3.485	0.712	−0.112 *	0.078	0.015	−0.044	0.207 ***	0.591 ***	1

Note: * *p* < 0.05, ** *p* < 0.01, *** *p* < 0.001. Square root of AVE presented along the diagonal Employer gender (male = 1; female = 2), Employer age (20–35 years old = 1; 36–50 years old = 2; 51 years old and above = 3), Employer education (Junior College and below = 1; Undergraduate = 2; Graduate and above = 3), Employer monthly income (below 8000 = 1; 8000–15,000 = 2; 15,000 yuan and above = 3), Gender of the leader (male = 0; female = 1), The age of the leader (20–35 years old = 1; 36–50 years old = 2; 51 years old and above = 3), The leader’s education (Junior College and below = 1; Undergraduate = 2; Graduate and above = 3), Team size (1–10 people = 1; 11–20 people = 2; 21 people and above = 3).

**Table 5:** The results of hypothesis testing.

Variables	Employee Pro-Environmental Behavior					Value–Action Barrier	
	Null Model 1	Model 1	Model 2	Model 3	Model 4	Null Model 2	Model 5
(Individual level)							
Intercept	3.320 ***	2.092	−0.339	2.009	0.239	3.760 ***	5.583 ***
Gender		0.058	0.058	0.069	0.068		0.043
Age		0.014	0.011	0.022	0.023		0.053
Educational level		−0.047	−0.042	−0.049	−0.047		−0.031
Monthly income		0.054	0.054	0.064	0.062		0.035
VAB				−0.241 ***	−0.252 ***		
(Team level)							
Gender		−0.032	−0.012	0.012	−0.078		0.086
Age		−0.345	−0.391	−0.409 *	−0.416 **		−0.065
Educational level		−0.182	−0.342	−0.404 *	−0.381 *		−0.171
Monthly income		0.075	−0.075	0.107	0.087		−0.132
Team size		0.522 ***	0.259	0.230	0.040		−0.105
GTL			0.506 ***	0.444 ***	0.407 ***		−0.266 ***
GBI					0.205 *		
GTL× GBI					0.466 ***		
GBI × VAB					0.359 **		
Statistics							
R (Sigma squared)	0.301		0.301	0.277	0.277	0.316	0.317
U (Tau)	0.510		0.113	0.101	0.067	0.415	0.367
Chi-square	49.927 ***	607.103 ***	538.480 ***	628.061 ***	581.231 ***	49.927 ***	581.231 ***
Deviance	1891.761	6250.812	5918.636	822.061	6087.836 ***	1891.761	6087.836 ***

Note: * *p* < 0.05, ** *p* < 0.01, *** *p* < 0.001.

**Table 6:** Mediation Analysis.

Path	Effect	Estimate	*p*	95% CI Lower Bound	95% CI Upper Bound
GTL → EPB	Direct effect	0.407	0.000	0.251	0.561
GTL → VAB → EPB	Indirect effect	0.066	0.000	0.024	0.121

**Table 7:** Moderating Effect of GBI on the Relationship between GTL and EPB.

GBI Level	Estimate	*p*	95% CI Lower Bound	95% CI Upper Bound
High GBI	0.873	0.000	0.647	1.102
Low GBI	−0.061	0.278	−0.262	0.140
Difference	0.932	0.000	0.635	1.247

**Table 8:** Moderating Effect of GBI on the Relationship between VAB and EPB.

GBI Level	Estimate	*p*	95% CI lower Bound	95% CI Upper Bound
High GBI	0.108	0.177	−0.123	0.333
Low GBI	−0.612	0.000	−0.838	−0.384
Difference	0.717	0.001	0.286	1.142

## Data Availability

Data will be accessible upon request.

## Supplementary Materials

The following supporting information can be downloaded at: https://www.mdpi.com/article/10.3390/bs16010071/s1, File S1: Informed Consent Form (Bilingual); File S2: Abbreviations and Questionnaire (English Translation).
