# Supplementary material for: Green Transformational Leadership and Value–Action Barrier on Employees’ Pro-Environmental Behavior: The Moderating Role of Green Brand Image in Chinese Food Manufacturing Enterprises
Source: Behav Sci (Basel). 2026 Jan 5;16(1):71. doi: 10.3390/bs16010071 (PMC12837736; doi:10.3390/bs16010071)
Supplement: Supplementary file 1 [file behavsci-16-00071-s001.zip › File S1. Informed Consent Form (Bilingual).pdf]

File S1. Informed Consent Form

Dear Participants,

Greetings! We are conducting an online survey on how green transformational leadership, value–action barriers, and green brand image influence employees' pro-environmental behavior. The survey is for academic purposes only. Thank you for your support. Please read the information below before participating.

I. Purpose and Background

This study examines the effect of green transformational leadership on employees' pro-environmental behavior, the mediating role of value–action barriers, and the moderating role of green brand image. Findings will inform both sustainability practice and academic research.

II. Questionnaire Content and Form

The questionnaire covers perceptions of leadership, organizational climate, and workplace green behaviors. It takes about **15–20 minutes**. Items are for research analysis only; there are no "right" or "wrong" answers. Please answer honestly.

III. Confidentiality and Data Protection

- No personally identifiable information (e.g., name, employer) is collected; data are anonymous and confidential.
- Data are used solely for academic research, stored securely, and destroyed after study completion.
- No personal information will be disclosed to third parties.

IV. Voluntary Participation and Withdrawal

Participation is entirely voluntary. You may skip any item and withdraw at any time without penalty or negative consequences.

V. Contact Information and Feedback

For questions, please contact:
[Researcher's Name]
Email: [Insert]
For concerns about participant rights, contact the [Institutional Review Board/Ethics Committee] at [Insert].

VI. Consent Statement

By checking below and submitting the survey, you confirm that you have read and understood the information above and voluntarily consent to participate.

□ I Agree　　□ I Do Not Agree

(For paper forms)
Name (optional): _____________
Signature: _____________
Date: ____//
