# Supplementary material for: Green Transformational Leadership and Value–Action Barrier on Employees’ Pro-Environmental Behavior: The Moderating Role of Green Brand Image in Chinese Food Manufacturing Enterprises
Source: Behav Sci (Basel). 2026 Jan 5;16(1):71. doi: 10.3390/bs16010071 (PMC12837736; doi:10.3390/bs16010071)
Supplement: Supplementary file 1 [file behavsci-16-00071-s001.zip › File S2. Abbreviations and Questionnaire.pdf]

File S2

1. List of Abbreviations

AVE	Average variance extracted
CFA	Confirmatory factor analysis
CFI	Comparative fit index
CR	Composite reliability
df	Degree of freedom
GTL	Green transformational leadership
EPB	Employees' pro-environmental behavior
VAB	Value-Action Barrier
GBI	Green brand image
GFI	Goodness of fit index
HLM	Hierarchical linear modeling
RMSEA	Root-mean-square error of approximation
SMEs	Small and medium-sized enterprises
SRMR	Standardized root mean square residual
TL	Tucker–Lewis index
x^2	Chi-square statistic

2. Questionnaire (English Translation)

Response format: Unless otherwise specified, all items were rated on a five-point Likert scale (1 = strongly disagree, 5 = strongly agree).

Administration (three-wave, time-lagged design):

At Time 1 (T1), employees rated GTL. At Time 2 (T2), employees rated VAB and team leaders rated team-level GBI. At Time 3 (T3), employees rated EPB.

A. Green Transformational Leadership (GTL) – Employee-rated (T1)

1.	My superiors actively promote the concept of environmental protection in daily management.
2.	My superiors encourage team member’ s to participation in green innovation initiatives.
3.	My superiors exemplify environmentally protective behaviors.
4.	My superiors often commend employees who excel in in environmental protection.
5.	My superiors establish explicit goals pertaining to environmental protection.
6.	My superiors prioritizes resource conservation and reutilization in their work.

B. Value-Action Barrier (VAB) – Employee-rated (T2)

1.	I regard environmental protection is crucial, yet I lack sufficient time to practice.
2.	Environmental protection actions require excessive energy.
3.	I aspire to protect the environment, but my peers do not endorse it.
4.	Inadequate environmental protection infrastructure hinders my ability to act.
5.	I’m concerned that environmental protection behavior may affect work performance.
6.	I believe my individual action have minimal impact on the environment.

C. Green Brand Image (GBI) – Team leader-rated (T2)

1.	I believe the company possesses a commendable reputation in environmental protection.
2.	The company's products demonstrates the commitment to environmental protection.
3.	The company is an industry leader in green production and packaging.
4.	The company actively communicates the concept of sustainable development to the public.
5.	I perceive the company as having a positive image regarding environmental protection.

D. Employees' Pro-Environmental Behavior (EPB) – Employee-rated (T3)

1.	I will proactively initiate efforts to minimize resource waste at work.
2.	I will actively endorse the company's environmental protection initiatives.
3.	I often remind my colleagues to conserve electricity and water.
4.	I am willing to try new environmental protection practices.
5.	I am open to experimenting with new environmental protection practices.
6.	I will propose enhancements to process aimed at pollution reduction.
7.	I am prepared to assume additional responsibilities for achieving the company's environmental objectives.

Note: The survey was administered in Chinese. The original questionnaire was developed in English, translated into Chinese, and back-translated by bilingual researchers to ensure linguistic consistency.
